# PHACCS, an online tool for estimating the structure and diversity of uncultured viral communities using metagenomic information

**DOI:** 10.1186/1471-2105-6-41

**Published:** 2005-03-02

**Authors:** Florent Angly, Beltran Rodriguez-Brito, David Bangor, Pat McNairnie, Mya Breitbart, Peter Salamon, Ben Felts, James Nulton, Joseph Mahaffy, Forest Rohwer

**Affiliations:** 1 Ecole Supérieure de Biotechnologie de Strasbourg, Boulevard Sébastien Brandt, 67413 Illkirch, France; 2 Department of Biology, San Diego State University, 5500 Campanile Drive, San Diego, California 92182, USA; 3 Department of Mathematics and Statistics, San Diego State University, 5500 Campanile Drive, San Diego, California 92182, USA; 4 Computational Science Research Center, San Diego State University, 5500 Campanile Drive, San Diego, California 92182, USA; 5 Center For Microbial Sciences, San Diego State University, 5500 Campanile Drive, San Diego, California 92182, USA

## Abstract

**Background:**

Phages, viruses that infect prokaryotes, are the most abundant microbes in the world. A major limitation to studying these viruses is the difficulty of cultivating the appropriate prokaryotic hosts. One way around this limitation is to directly clone and sequence shotgun libraries of uncultured viral communities (i.e., metagenomic analyses). PHACCS , Phage Communities from Contig Spectrum, is an online bioinformatic tool to assess the biodiversity of uncultured viral communities. PHACCS uses the contig spectrum from shotgun DNA sequence assemblies to mathematically model the structure of viral communities and make predictions about diversity.

**Results:**

PHACCS builds models of possible community structure using a modified Lander-Waterman algorithm to predict the underlying contig spectrum. PHACCS finds the most appropriate structure model by optimizing the model parameters until the predicted contig spectrum is as close as possible to the experimental one. This model is the basis for making estimates of uncultured viral community richness, evenness, diversity index and abundance of the most abundant genotype.

**Conclusion:**

PHACCS analysis of four different environmental phage communities suggests that the power law is an important rank-abundance form to describe uncultured viral community structure. The estimates support the fact that the four phage communities were extremely diverse and that phage community biodiversity and structure may be correlated with that of their hosts.

## Background

Most environmental viruses are phages (a.k.a., bacteriophages) that infect prokaryotic cells, both Bacteria and Archaea. On average there are about ten phage particles per host cell [1]. Extrapolations from the number of prokaryotes [2] make phages the most abundant biological entities in the biosphere with an estimated 10^31 ^viral particles. By killing prokaryotes, phages can strongly impact microbial community biomass [3] and structure [4]. Despite their importance, very little is known about phage biodiversity.

Traditionally, the study of environmental phage diversity, dynamics, and ecology requires growing prokaryotes on microbiology plates and infecting them with phages. However this standard technique is limited by the fact that only a small fraction of environmental microbes are readily cultured [5] and that each phage species generally only has a very narrow number of possible microbial hosts [6]. In addition, even if it is possible to observe phages with an electron microscope, pictures are not sufficient to identify species because of the low taxonomic resolution of viral morphology. Cultivating and observing phages do not permit to assess environmental phage diversity.

Biodiversity is composed of richness, or total number of different species [7], and evenness, expressing the relative abundance of each species [8]. The Shannon-Wiener index quantifies diversity as a single term combining richness and evenness [9]. A high richness and high evenness together represent a high level of diversity.

A new approach to accessing natural microbial diversity is through the creation of shotgun sequence libraries from environmental metagenomes (sum of all genomes) [10-14], so that the genetic information of each genotype of the community is recorded, qualitatively (sequence) and quantitatively (abundance of each sequence). The community is analyzed by sequencing a part of the library. The metagenomic data used here is the contig spectrum, determined by assembly of environmental random shotgun DNA fragments. The contig spectrum is a vector containing the number of contigs (groups of overlapping sequences) of size *q *(number of sequences in the group) [10]. The stringency of the assembly parameters can be varied so that only sequences belonging to the same genotype overlap. Thus, for one genotype, the bigger the contigs in the contig spectrum, the higher the number of copies and the more abundant this genotype. Based on this, the contig spectrum provides important information about the abundance and diversity of genotypes within a community.

In this work, we present PHACCS (PHAge Communities from Contig Spectrum), an online computational tool to assess the diversity and structure of environmental viral communities from the contig spectrum of shotgun sequence data. The PHACCS program and its predictions are first described and then used to analyze four environmental viral communities.

## Implementation

### Platform and software

The standalone core mathematics for PHACCS consists of Matlab (MathWorks Inc., Natick, MA.) scripts that are partly based on the previous works [10-12]. A CGI (Common Gateway Interface) script written in PERL (Practical Extraction and Report Language) is used to input and output data from and to an HTML (Hyper Text Markup Language) interface. PHACCS was developed and tested on a Linux-based (2.6.6 kernel) personal computer running PERL 5.8.3 (with CGI module), Matlab 6.5.0, and Apache 2.0.50 web server.

### Obtaining a contig spectrum

The input for PHACCS is the contig spectrum, a vector containing the number of *q*-contigs (groups of *q *overlapping sequences) from the *in silico *assembly of random shotgun DNA fragments. Detailed information about the way to get viral metagenomes and their contig spectrum can be found in [10-12]. Briefly, viral communities were isolated via tangential flow filtration and cesium chloride centrifugation, and their DNA was extracted. The DNA was randomly fragmented, used to create a linker amplified shotgun library [15] and clones were sequenced (between 500 and 1200 for studies [10-12]). The sequence assembly program Sequencher (Gene Codes Corp., Ann Arbor, MI.) was used to assemble phage sequences having at least 98% identity on at least 20 bp [10]. The stringency of the assembly parameters was experimentally determined so that only fragments belonging to the same genotype assemble together. Closely related phage genomes (e.g., coliphages T3 and T7) can be discriminated using these parameters [10]. The number of contigs of each size was then recorded to generate the contig spectrum. The number of sequences in the largest contig defines the contig spectrum degree.

### Modified Lander-Waterman algorithm

PHACCS uses a modified version of the Lander-Waterman algorithm [16] to predict a contig spectrum from assumed population parameters. The original Lander-Waterman algorithm is a way of predicting the contig spectrum of a randomly fragmented genome (e.g., a single viral species) given: i) the length *L *of the genome, ii) the number *N *of DNA fragments studied, iii) the average size *s *of these fragments, and iv) the minimum overlap length *o *for the sequence assembly [16]. Given this data, the predicted values of the following quantities are calculated:

• Probability *p *of an overlap: *p *= 1 - *e*^-*Nx*/*L *^with *x *= *s *- *o*

• Probability *w*_*q *_for a fragment to be part of a *q*-contig (overlap of *q *fragments):

*w*_*q *_= *qp*^*q *- 1 ^(1 - *p*)^2^

• Expected number of fragments *c*_*q *_that are part of a *q*-contig: *c*_*q *_= *Nw*_*q*_

• Contig spectrum: 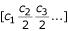


The modified Lander-Waterman algorithm is a generalization of the original algorithm to a group of *M *different genotypes (e.g., a whole viral community) [10]. The predicted contig spectrum can be calculated as the sum of the contig spectra for each individual genotype *i*.

• Expected number of fragments *c*_*q *_part of a *q*-contig:


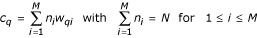


*w*_*qi *_is the probability for a fragment to be part of a *q*-contig for the genotype *i *and *n*_*i *_is the expected number of fragments for the genotype *i*.

In this modified algorithm, since there are several genotypes, an assumption about their underlying distribution within the community in terms of abundance has to be made.

### Relative rank-abundance forms

PHACCS offers six basic functional forms of relative rank-abundance for biological populations: the power law, logarithmic, exponential, broken stick, niche preemption, and lognormal distributions.

The first three functional forms are empirical models that were designed to describe an asymptotic drop-off in the abundance [17]:

• Power: *n*_*i *_= *ai*^-*b *^for 1 ≤ *i *≤ *M*

• Logarithmic: *n*_*i *_= *a*(log(*i *+ 1))^-*b *^for 1 ≤ *i *≤ *M*

• Exponential: *n*_*i *_= *ae*^-*ib *^for 1 ≤ *i *≤ *M*

The parameter *a *represents the abundance of the most abundant genotype, *b *is a parameter related to the evenness, and *M *is the number of different genotypes in the community.

Two ecological models are based on a partitioning of resources between species [18,19]:

• Broken stick: 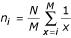 for 1 ≤ *i *≤ *M*

• Niche preemption: *n*_*i *_= *Nk*(1 - *k*)^*i *- 1 ^and *n*_*M *_= *N*(1 - *k*)^*M *- 1 ^for 1 ≤ *i *≤ *M *- 1

The broken stick function has only one parameter, *M*, and assumes a random distribution of resources, whereas in the niche preemption function, each species takes only a fraction *k *of the remaining resources in the environment.

The sixth functional form is the lognormal distribution. It is the most commonly used species distribution, with numerous theoretical justifications in the literature [20,21]. The relationship is specified as species density versus abundance and needs to be transformed to give a rank-abundance relationship. Our rank-abundance form was obtained by dividing the area under the normal distribution with standard deviation *σ *into *M *equal area slices and associating an abundance *n*_*i *_with the *i*-th slice by calculating an average value for the abundance within the slice. The result is:


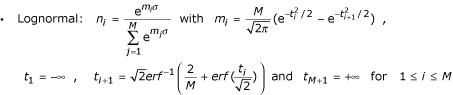


where *erf *is the error function and *erf*^1 ^its inverse.

### Modeling the viral community structure

**Figure 1:**
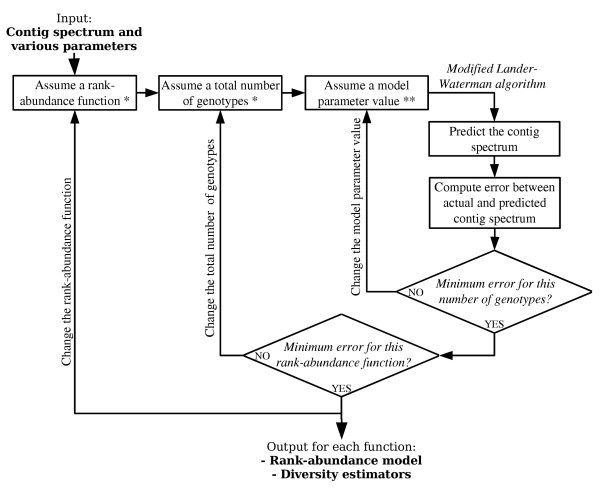
Flowchart of PHACCS. *The rank-abundance functions and the range of genotypes to use can be defined by the user. **This parameter represents b for the power law, logarithmic, lognormal and exponential distributions and k for the niche preemption. This parameter is not applicable to the broken stick.

The PHACCS algorithm is represented in Figure 1. The experimentally determined contig spectrum of a sample and the other parameters needed for the modified Lander-Waterman algorithm are the input. For a given rank-abundance function, assumed values of the function parameters (number of different genotypes, as well as *b *for the power law, logarithmic, exponential and lognormal distributions and *k *for niche preemption) are used to predict a contig spectrum using the modified Lander-Waterman algorithm. To determine the model fitness, the error between the actual and the predicted contig spectrum is calculated as the variance-weighted sum of squared deviations, *L *being the contig spectrum vector length and *c*_*q*_' the experimental number of fragments that belong to a *q*-contig:


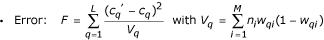


The best descriptive model for a community structure is defined as the one with the smallest error. For each rank-abundance function tested, the global minimum for the error is found by optimizing the value of the function parameters.

The values of the error can be roughly interpreted as logarithms of odds ratios of the observed contigs being seen from community distributions of the specified forms. Thus a value of 0.1 for the difference in errors between two models corresponds to an odds ratio of e^0.1 ^which is about 11:10 between the two models. This means that the model with the smallest error is about 10% more likely to give rise to the observed data.

### Predicting the viral community diversity

For each rank-abundance form, the best model is used by PHACCS to assess diversity. The richness *S *is estimated as equal to the number of different genotypes *M *found in the community structure model. The abundance of the most abundant genotype is also directly determined from the model as the highest rank-abundance value. The Shannon-Wiener index, which is a measure for diversity, is calculated using the relative rank-abundance values *r*_*i *_= *n*_*i*_/*N *of all individual genotypes *i *[9]:

• Shannon-Wiener index *H*' (in nats): 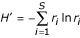


The evenness is derived from *H*' [18]:

• Evenness *E*: *E *= *H*'/*H*_*max *_= *H*'/ln *S*

### Comparison of four phage communities

As a case study, four viral metagenomes obtained from previous studies and belonging to different ecosystems were tested. Two of these were phage community samples of near-shore surface seawater from Scripps Pier (SP) and Mission Bay (MB), San Diego, California, USA [10]. The two other samples are sediments from Mission Bay (MBSED) [11] and human feces (FEC) [12]. A compilation of the data for these samples is presented in Table 1. These four datasets were analyzed with PHACCS using all six rank-abundance models.

## Results

### Best abundance forms

The errors obtained from the contig spectrum analysis of the different samples are presented in Table 2. For each sample the best descriptive model of the community structure is the one with the smallest error. The SP community was best described by using the power law (error of 1.84), closely followed by the lognormal (error of 1.93) and logarithmic (error of 2.57) distributions. The exponential and niche preemption distributions had poor fits, with errors of 12.0. The MB community modeling gave qualitatively the same results. Power law was the best fit with an error of 2.15 and exponential and niche preemption were last with an error of 16.2. The FEC community also had the same sequence of best fitting rank-abundance forms. The best model was given by using the power law form (error 9.79). Exponential and niche preemption did a poor job of explaining the data, coming in last with an error of 60.0. For the MBSED community, the power law, lognormal, logarithmic and exponential distributions all tied for the best fit (with an error of 0.0104), whereas broken stick gave the worst fit (error of 0.0157).

### Phage community diversity and structure

**Figure 2:**
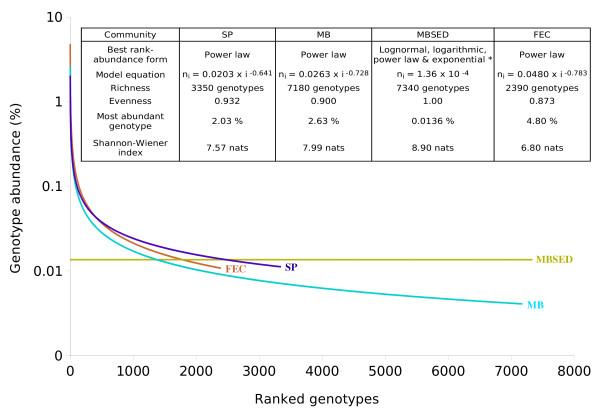
Comparison of the structure and diversity of the different viral communities using PHACCS. The graphics represent rank-abundance curves, where the abundance of each genotype is plotted versus its abundance rank, the genotype of rank one being the most abundant. The curves were obtained by plotting the PHACCS rank-abundance values of the different communities on the same axis. *The predicted community structure for MBSED was the same for the lognormal, logarithmic, power and exponential rank-abundance forms. As a consequence, the diversity predictions were also the same.

The different diversity indicators and the rank-abundance curves obtained by using the best descriptive model for each sample are summarized in Figure 2. The MBSED community was the richest with an estimated 7340 different phage genotypes. MB had ~7180 different genotypes, SP ~3350, and FEC was the least rich sample with ~2390 different genotypes. MBSED was the most even community with the maximum possible evenness of 1.00 (flat rank-abundance curve), followed by SP (evenness of 0.932), MB (evenness of 0.900), and FEC (evenness of 0.873). The most abundant genotype represented 4.80% of the total community for FEC, 2.63% for MB, 2.03% for SP and around 0.01% for MBSED. Based on the Shannon-Wiener diversity index, MBSED was overall the most diverse community with 8.90 nats, then MB (7.99 nats), SP (7.57 nats), and finally FEC (6.80 nats), the least diverse community.

## Discussion

### Using PHACCS

**Figure 3:**
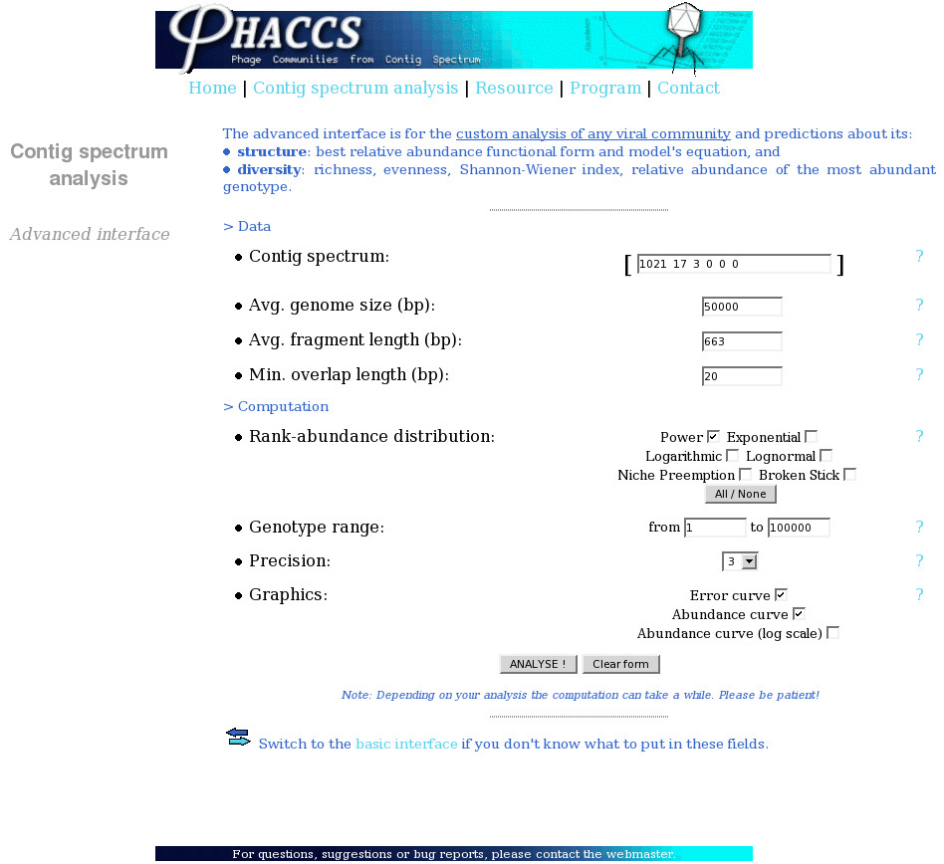
Screenshot of PHACCS' advanced web interface.

PHACCS is publicly accessible at  and the source code is freely available [see Additional file 1]. The biological information PHACCS needs as an input is the viral community's contig spectrum, average genome size, average shotgun DNA sequence length, and the minimum overlap length used for the assembly. PHACCS has two HTML interfaces. The basic interface assumes default values for marine phage communities (average genome size of 50 kb, average fragment length of 650 bp and minimum overlap of 20 bp). All rank-abundance forms (power law, expoential, logarithmic, lognormal, broken stick and niche preemption distributions) are tested for up to 100,000 genotypes. In the advances interface (Figure 3) the user can change all biological and computational parameters.

PHACCS analyses are computer intensive. On a dual-Opteron™ server, the computation for the SP sample takes ~5 minutes. The broken stick and lognormal rank-abundance forms account for most of the computation time (data not shown). Increasing the range of genotypes to search dramatically increases the time needed to complete the analysis (data not shown).

PHACCS estimations about the virus community are: i) structure – best descriptive rank-abundance form, model equation and error, and ii) diversity – richness, evenness, abundance of the most abundant genotype, and Shannon-Wiener index. Graphic representations of the community structure and of the error minimization can also be displayed. The error provides information about which model has the best fit relative to the others for a given contig spectrum. For each type of distribution, the user is informed if the best model (i.e., the error's global minimum) has not been found using the given computation parameters.

### Importance of the contig spectrum quality

Predictions by PHACCS are dependent on the quality of the contig spectrum input. The difference in error between two models can be small (Table 2) and using an inappropriate model can change the estimated diversity. For example, the predicted richness for the SP sample is about four times higher for the lognormal distribution than for the power law (data not shown). A useful contig spectrum requires that: i) the same clone be sequenced only once (remove all redundant clones), ii) the sequences be trimmed to remove ambiguities ("N"'s) and, iii) the assembly parameters be sufficiently stringent so that only sequences from the same genotype are part of the same contig (experimental determination by assembly of known sequences). All these experimental problems bias the observed occurrence of the DNA fragments, and thus the contig spectrum. Additionally, accurate community estimations are not to be expected if the contig spectrum only has a small degree (only small contigs) (e.g., MBSED, [1152 2 0 ...]). As a general rule, the higher the contig degree, the better the estimations, because the model fitting is done over a larger number of points. For the same reason, the number of trailing zeros in the contig spectrum is important. Adding zeros at the end of the contig spectrum will improve PHACCS predictions (e.g., 10 trailing zeros were used in the present analyses) but will also increase the computation time.

### Limitations

The way the contig spectrum is obtained leads to approximations of the viral diversity. In the samples analyzed here, only the DNA from viruses smaller than 0.22 *μ*m is collected. Larger viruses and RNA viruses are not represented in the shotgun library and in the resulting contig spectrum. The contig spectrum assembly parameters (98% identity on at least 20 bp for phages) are stringent enough to limit the number of false-contigs (contigs between DNA fragments from different genotypes), but may on the other side omit some true-contigs (DNA fragments that are designated as non-overlapping when they actually belong to the same genotype). Additionally, the present implementation of the Lander-Waterman algorithm assumes that all DNA fragments and all the genotypes have the same size. For these reasons, PHACCS estimates should be considered approximations.

### Phage community structure and diversity

The comparative analysis of the four phage communities showed that the power law seems overall to be a powerful rank-abundance distribution to model phage community structure (Figure 2). A recent simple predator-prey model based on the observed marine phage-host dynamics explains how a power law distributed phage rank-abundance can be obtained from a modified Lokta-Volterra model [23]. Before analyzing the viral samples with the contig spectrum approach, the number of viral genotypes in an environment was totally unknown. The viral communities turned out to be extremely diverse with estimated Shannon-Wiener diversity indices between 6.8 nats (fecal sample) and 8.9 nats (sediment sample) (Figure 2), representing diversity levels higher than for most bacterial communities [11]. Because phages are specific predators, the structure and diversity of phage communities could be directly correlated to the structure and diversity of the coexisting microbial communities [2]. Some facts seem to support this hypothesis.

First, the extreme diversity of the sediment viral community may reflect the higher diversity of the microbial communities found in sediments using automated rRNA intergenic spacer analysis (ARISA) [24] in comparison with seawater. Also, only a few hundred different bacterial species were reported in the human colon intestinal flora [25] using the 16s ribosomal DNA methodology, which could account for the relatively low phage richness in the fecal sample.

## Conclusion

PHACCS is a web-based service that predicts community structure and diversity using the contig spectrum from metagenomic random shotgun sequence data. This methodology allows PHACCS to determine the mathematical model that most accurately reflects the underlying genotype abundance distribution (i.e., power law, logarithmic, exponential, broken stick, niche preemption, or lognormal distributions) and use it to makes estimates about the diversity of the communities, (i.e., richness, evenness, Shannon-Wiener index and abundance of most abundant genotype). Using uncultured environmental viral samples, PHACCS has been used to confirm that phage biodiversity is higher than in any previously observed community and that the structure of viral communities may closely follow that of their hosts. PHACCS is designed for biologists to mathematically analyze their viral shotgun libraries and gain insights about viral ecology and population dynamics.

## Availability and requirements

• **Project name: **PHACCS – PHAge Communities from Contig Spectrum

• **Project home page: **

• **Operating system(s): **Unix based system for PHACCS and its web interface. Platform independent for PHACCS core.

• **Programming language: **Matlab (for the core scripts) and Perl

• **Other requirements: **For the interface: CGI.pm Perl module, ppmtogif, webserver program (to use PHACCS as a web service)

• **License: **GNU GPL

## Authors' contributions

FA developed the PHACCS main program and its interface. BRB helped with the programming. BRB, DB, PMN, PS, BF, JN and JM developed the modified Lander-Waterman algorithm and implemented it with Matlab. FR and MB helped write the manuscript and provided the test datasets. All authors read and approved the final manuscript.

## Supplementary Material

Additional File 1 This file contains the script files part of PHACCS. These files are either standard text or picture files. Click here for file

## Figures and Tables

**Table 1:** Test data used for the study of the phage communities with PHACCS [10-12]. The average fragment sequence length was determined using Sequencher after sequence trimming (maximum one ambiguity on 99 bp at each extremity). A 98% identity for a minimal overlap length of 20 bp was used for sequence assembly with Sequencher to obtain the contig spectra. The average genome length was determined by pulse field gel electrophoresis [12, 22].

**Community**	**SP (Scripps Pier)**	**MB (Mission Bay)**	**MBSED (Mission Bay Sediments)**	**FEC (Fecal)**
Contig spectrum *	1021 17 2 0 ...	841 13 2 0 ...	1152 2 0 ...	482 18 2 2 0 ...
*Avg. community genome size*	50 kb	50 kb	50 kb	30 kb
*Avg. shotgun fragment length*	663 bp	663 bp	570 bp	699 bp

* The number of trailing zeros was set to 10 for each contig spectrum.

**Table 2:** Best descriptive rank-abundance form for the viral communities as determined by PHACCS. The error represents the variance weighted sum squared deviation between the experimental and the predicted contig spectra. For each community, the best descriptive function is the one that minimizes the error. The best fit obtained for each rank-abundance form was ranked according to the error in ascending order.

SP			MB			MBSED			FEC		
*Rank*	*Model*	*Error*	*Rank*	*Model*	*Error*	*Rank*	*Model*	*Error*	*Rank*	*Model*	*Error*

1	**Power law**	**1.84**	1	**Power law**	**2.15**	1	**Power law**	**0.0104**	1	**Power law**	**9.79**
2	Lognormal	1.93	2	Lognormal	2.36	1	**Lognormal**	**0.0104**	2	Lognormal	10.2
3	Logarithmic	2.57	3	Logarithmic	2.88	1	**Logarithmic**	**0.0104**	3	Logarithmic	10.3
4	Broken stick	10.7	4	Broken stick	14.6	1	**Exponential**	**0.0104**	4	Broken stick	52.2
5	Exponential	12.0	5	Exponential	16.2	5	Niche preemption	0.0139	5	Exponential	60.0
5	Niche preemption	12.0	5	Niche preemption	16.2	6	Broken stick	0.0157	5	Niche preemption	60.0
