# Supplementary material for: PHACCS, an online tool for estimating the structure and diversity of uncultured viral communities using metagenomic information
Source: BMC Bioinformatics. 2005 Mar 2;6:41. doi: 10.1186/1471-2105-6-41 (PMC555943; doi:10.1186/1471-2105-6-41)

PHACCS

Phage Communities from Contig Spectrum

A computational tool for estimating the structure and diversity of uncultured viral communities using metagenomic information

Florent Angly

SDSU

San Diego State University

esbs

Ecole supérieure de biotechnologie Strasbourg

Ecole Supérieure de Biotechnologie de Strasbourg

Angly F., Brito B. R., Salamon P. , Rohwer F. 2004. PHACCS, a computational tool for estimating the structure and diversity of uncultured viral communities using metagenomic information. Manuscript in preparation...

The importance of phages

- Phage = virus infecting a prokaryote (a.k.a., bacteriophage)

- Most abundant biological entities on earth: ~10^{31}

- Ratio of 10 phages:1 prokaryote!

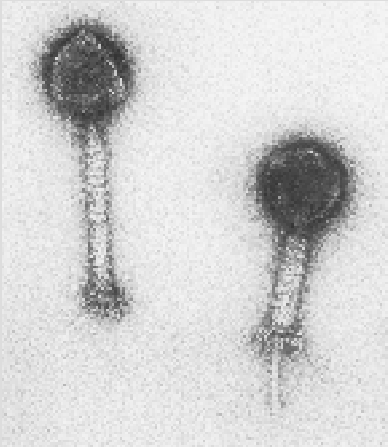

Phage TEM image from : http://www.ai.mit.edu/.../microbial-engineering.html

- Strong impact on microbial communities --> predation

- Little is known about phage ecology, diversity, etc ... and few tools to study them are available!

Goals of PHACCS

- PHACCS uses a metagenomic information:

  contig spectrum

- PHACCS gives an estimation of:

DIVERSITY → *Richness*

DIVERSITY → *Evenness*

DIVERSITY → *Shannon-Wiener index*

STRUCTURE → *Rank-abundance model*

Obtaining a contig spectrum

1 Metagenomic phage DNA from environmental sample

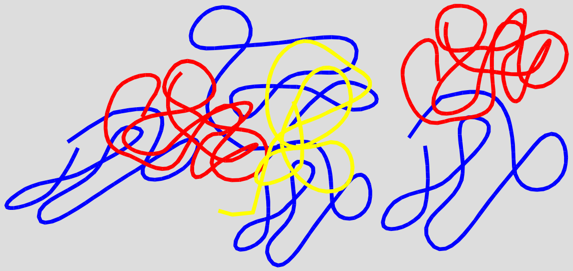

2 Fragment DNA randomly

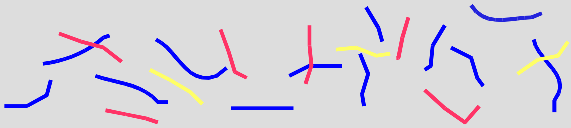

3 Clone fragments and sequence a lot of them

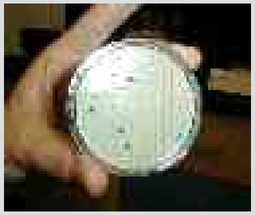

Shotgun sequencing

4 Assemble sequences

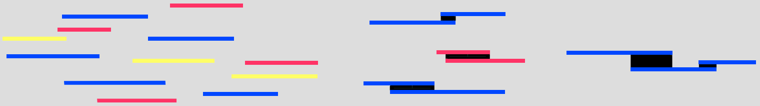

1-contigs *2-contigs* *3-contigs*

5 Calculate contig spectrum

[12 3 1 0 0 ...]

Contig spectrum assumption and signification

- **Only fragments from the same genotype should assemble together.**

  > overlap of minimum 20bp with 98% identity

- **For a given genotype, the bigger the contigs, the more abundant this genotype.**

  > contig spectrum ~ diversity

Original Lander-Waterman algorithm

<u>For ONE genotype:</u>

Genotype of length L: 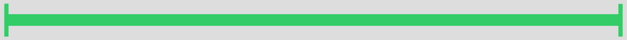

↓

n randomly cut fragments (average size s): 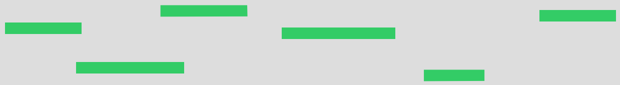

↓

Probability of an overlap of o bp: $p = 1 - e^{-nx/L}$ ($x = s$-o) 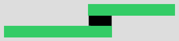

↓

Probability of a contig of q sequences: $w_q = qp^{q\;\;1}(1-p)^2$ 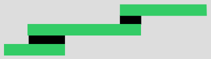

↓

Expected contig spectrum: $c_q = nw_q$ [c1 c2 c3 ...]

Lander, E. S., Waterman M. S. 1988. Genomic mapping by fingerprinting random clones: a mathematical analysis. Genomics. 2(3):231-239.

Modified Lander-Waterman algorithm

For SEVERAL genotypes:

Under an assumed distribution

M different genotypes of average length L (metagenome)

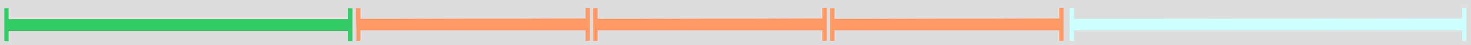

Community contig spectrum
=
sum of all individual contig spectra

$$c_q = \sum_{i=1}^{M} n_i w_{qi}$$

Breitbart M., Salamon P., Andresen B., Mahaffy J.M., Segall A.M., Mead D., Azam F., Rohwer F. 2002. Genomic analysis of uncultured marine viral communities. Proc Natl Acad Sci. 99(22):14250-5.

Analyzing the contig spectrum

Input: **contig spectrum**

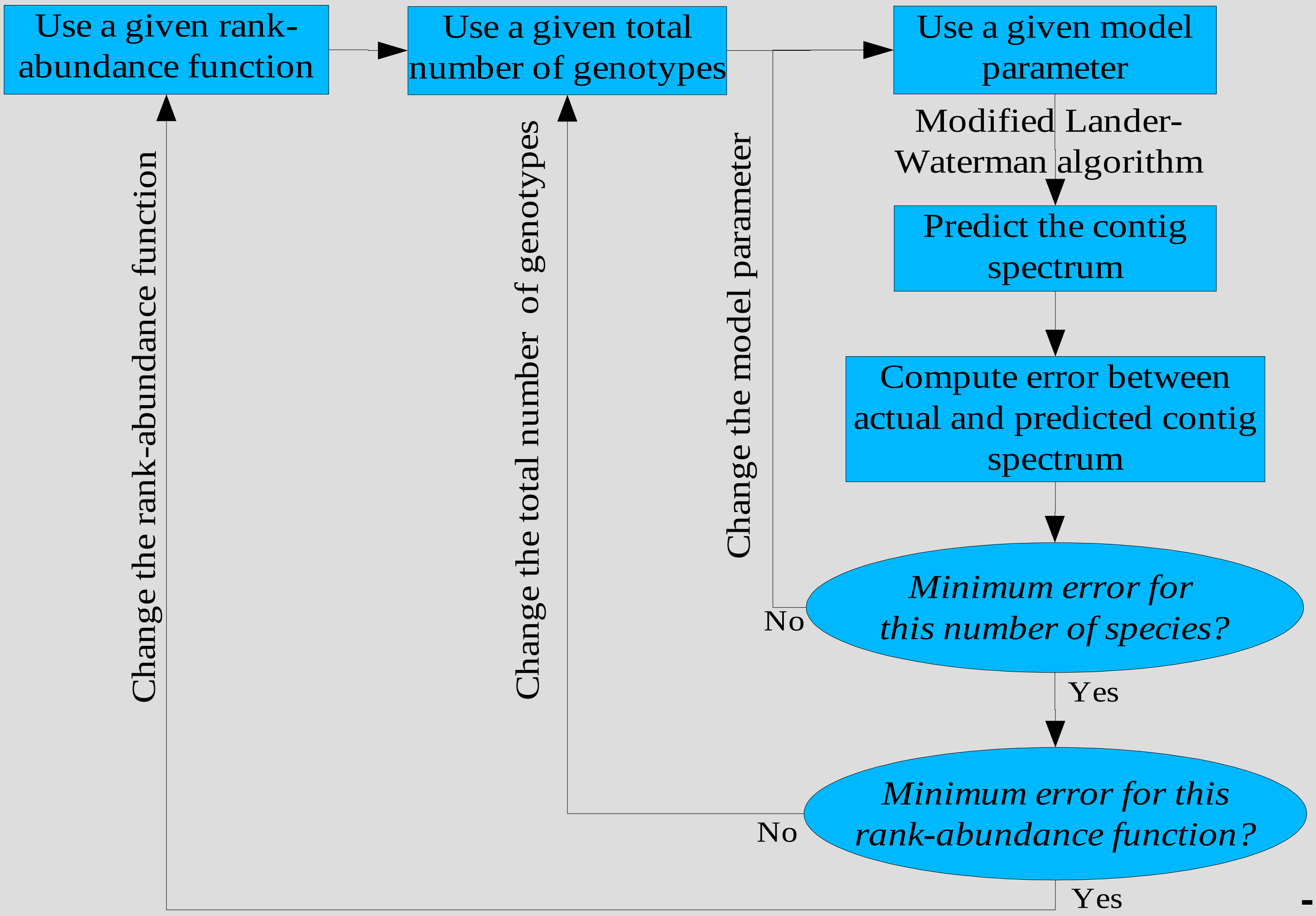

Output:

- rank-abundance model

- diversity estimators

Testing different community models

- 6 abundance functions:
  Power, Exponential, Logarithmic, Lognormal, Niche preemption & Broken stick law

- Rank-abundance distribution:

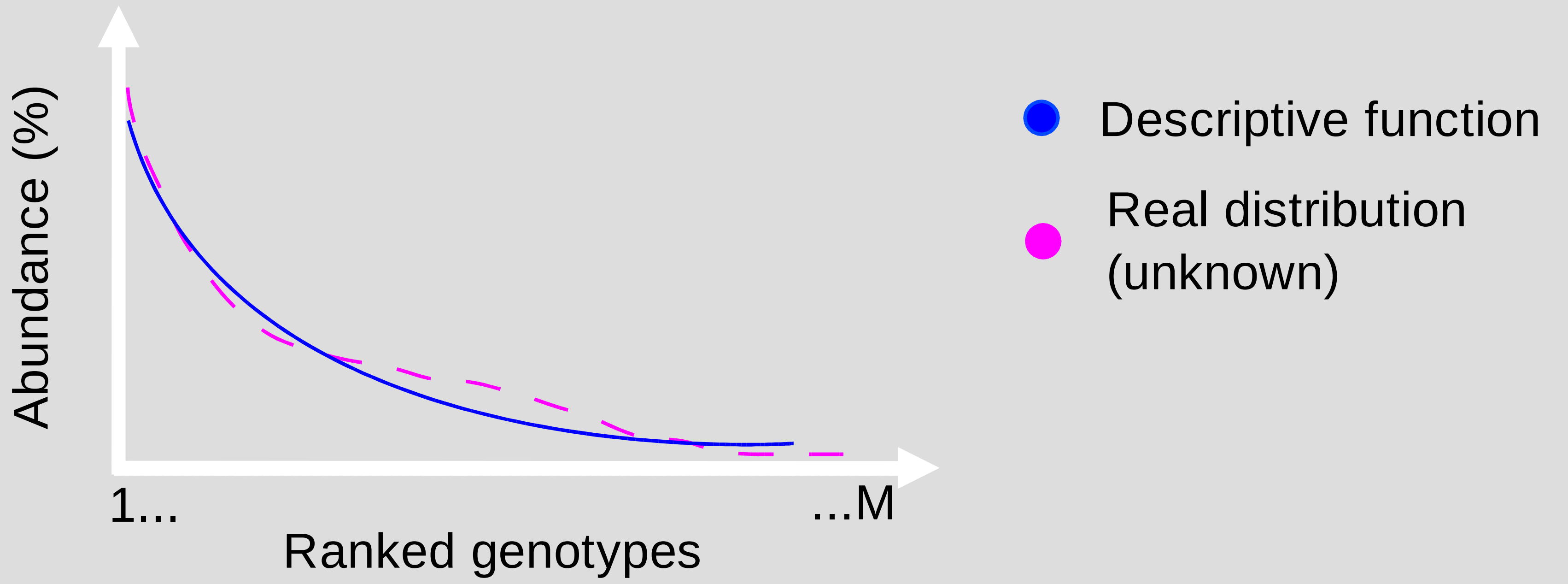

Making PHACCS accessible online

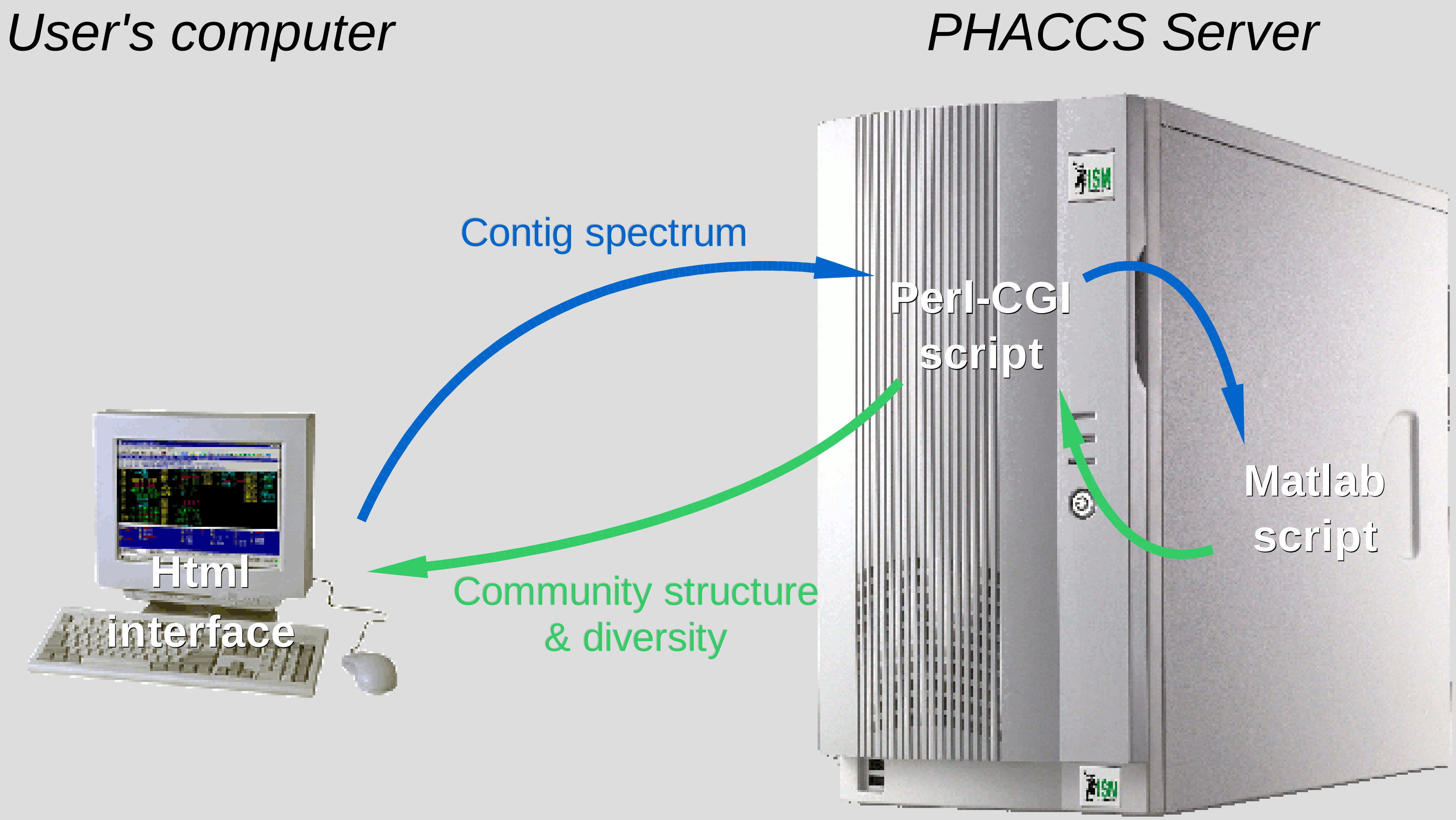

PHACCS online

http://phage.sdsu.edu/phaccs/

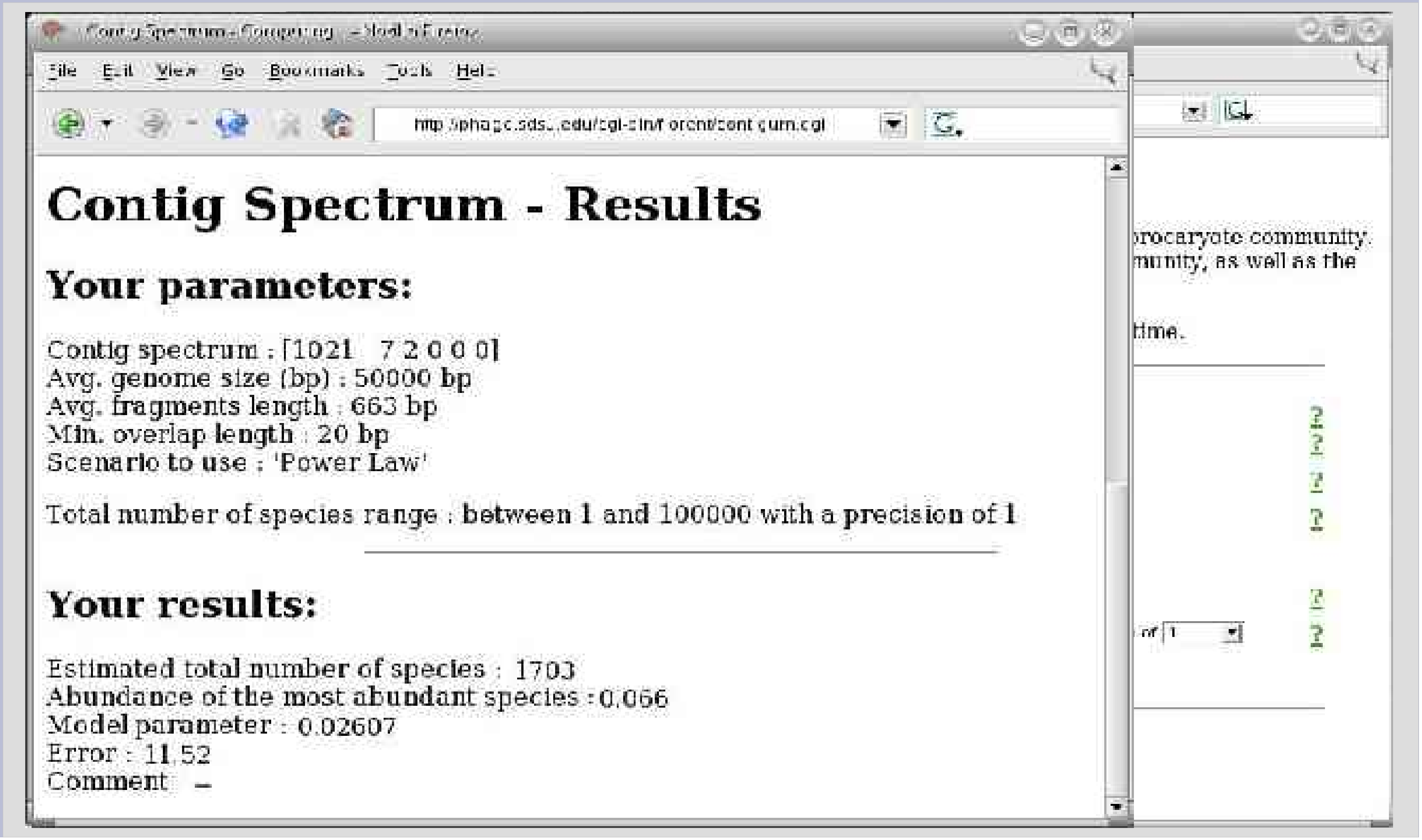

Case study

- 4 different phage communities from San Diego (USA):

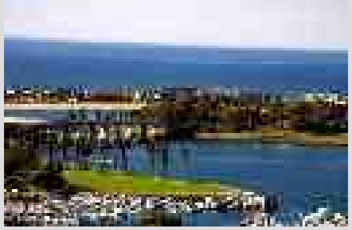

MB: surface seawater from Mission Bay
MBSED: sediments from Mission Bay

SP: surface seawater from Scripps Pier

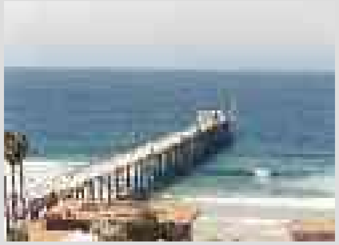

FEC: human feces

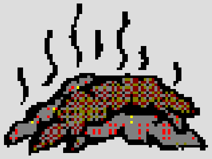

>>> What is structure of these communities?

>>> How diverse are they?

Breitbart et al. 2002. Proc. Natl. Acad. Sci. 99(22):14250-5.
Breitbart et al. 2003. J. Bact. 185(20):6620-6223.
Breitbart et al. 2004. Proc. R. Soc. Lond. 271:565-574.

Case study

Best descriptive function

Community	SP		MB		MBSED		FEC	
Model rank	*Model*	*Error*	*Model*	*Error*	*Model*	*Error*	*Model*	*Error*
1	**Power**	**1.81**	**Power**	**2.11**	**Power, Lognormal, Logarithmic & Exponential**	**0.0104**	**Logarithmic**	**8.81**
2	Lognormal	1.90	Lognormal	2.31			Power	8.87
3	Logarithmic	2.51	Logarithmic	2.81			Lognormal	9.01
4	Broken stick	10.7	Broken stick	14.6			Broken stick	52.2
5	Niche preemption & Exponential	12.0	Niche preemption & Exponential	16.2	Niche preemption	0.0139	Niche preemption & Exponential	60.0
6					Broken stick	0.0157		

On the average, power law seems to be the best function to model the structure of phage communities.

Case study

Community structure and diversity

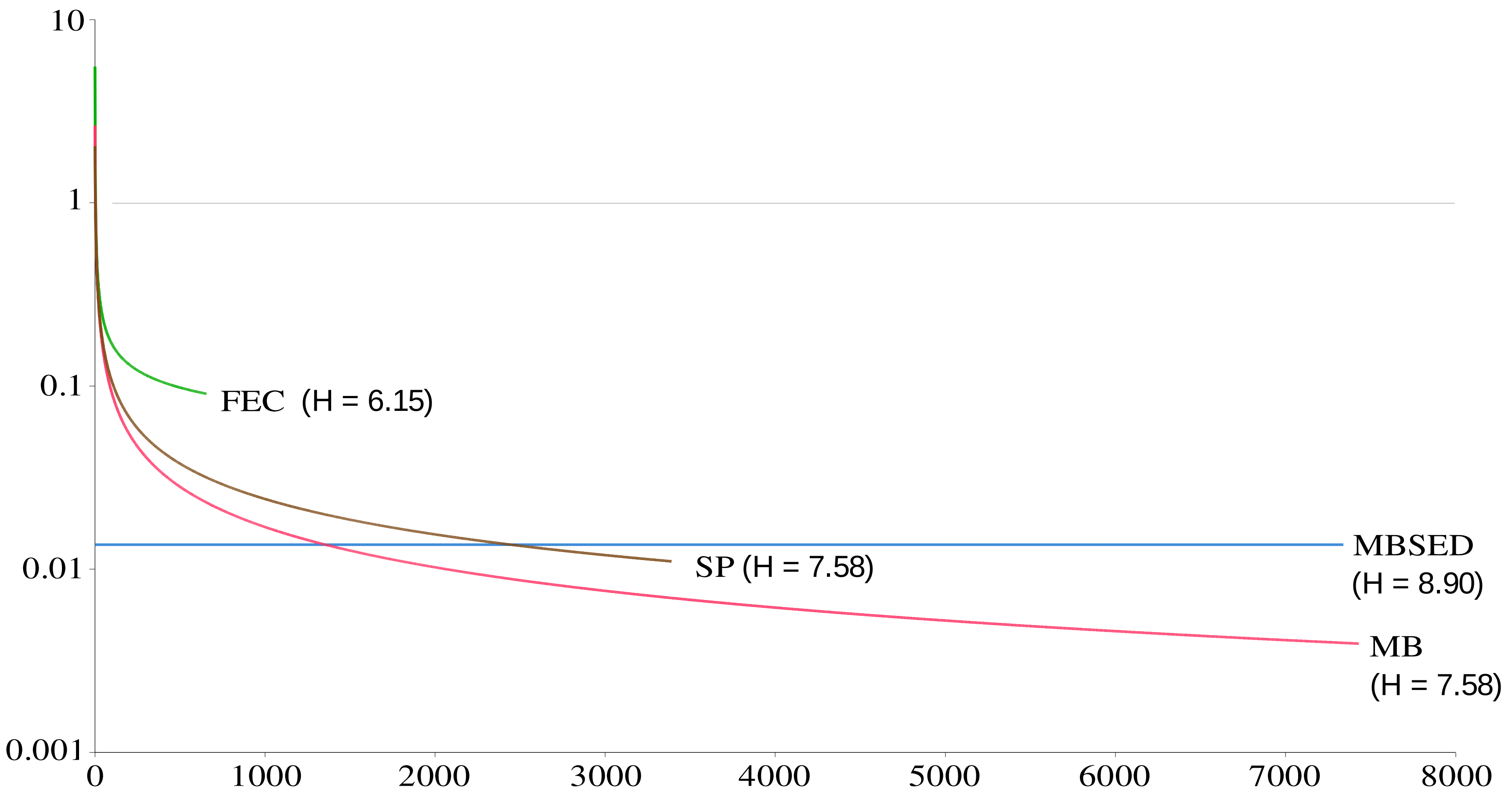

Conclusion

- PHACCS is web-based tool for analyzing the diversity and structure of uncultured viral communities.
- Advantages:
  - Estimation of viral diversity, what no simple experiment can do
  - Use of contig spectrum (metagenomic data) to better assess diversity
  - Web-based with a convenient interface
- PHACCS will help biologists to mathematically study their shotgun libraries and to have insight about phage ecology and interactions with prokaryotes communities.

PHACCS
Phage Communities from Contig Spectrum

- Thank you for your attention!
- Thanks to Forest and all the lab members!

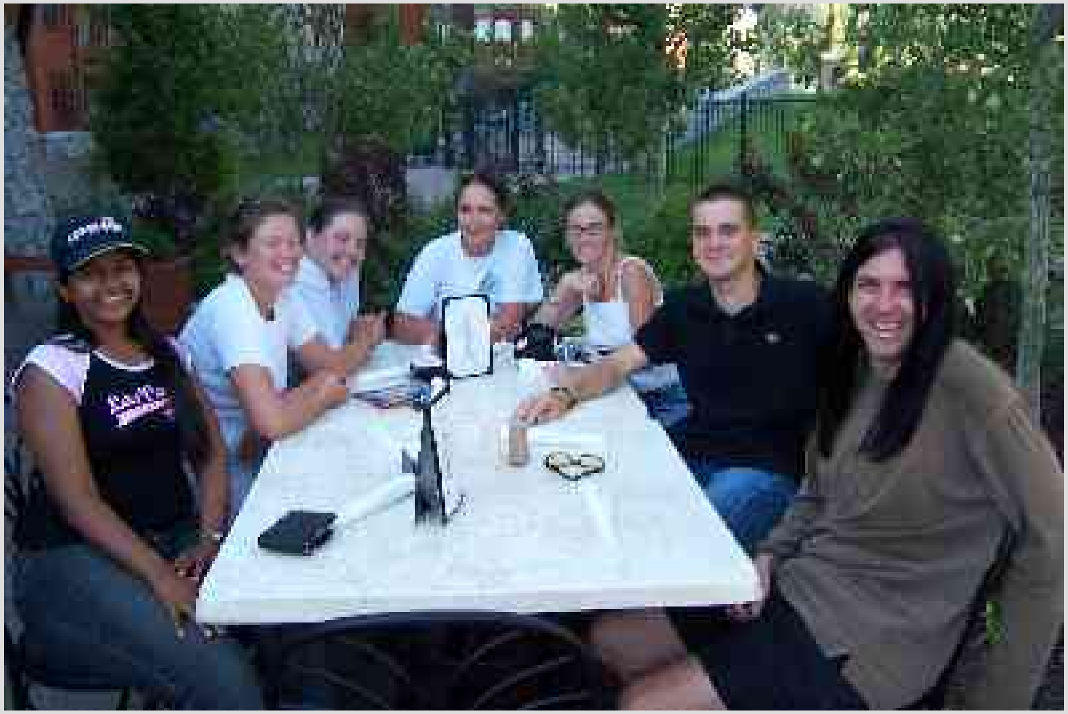

Supplement: Additional File 1 — This file contains the script files part of PHACCS. These files are either standard text or picture files. [file 1471-2105-6-41-S1.zip › PHACCS_V101/html/phaccs/data/PHACCS_presentation.pdf]
