# Supplementary material for: PHACCS, an online tool for estimating the structure and diversity of uncultured viral communities using metagenomic information
Source: BMC Bioinformatics. 2005 Mar 2;6:41. doi: 10.1186/1471-2105-6-41 (PMC555943; doi:10.1186/1471-2105-6-41)
Supplement: Additional File 1 — This file contains the script files part of PHACCS. These files are either standard text or picture files. [file 1471-2105-6-41-S1.zip › PHACCS_V101/html/phaccs/results/model-results-7121814798.htm]

Contig spectrum analysis results


|  |  |  |  |  |  |  |  |  |  |  |  |  |  |  |  |  |  |  |  |  |  |  |  |  |  |  |  |  |  |  |  |  |  |  |  |  |  |  |  |  |  |  |  |  |  |  |  |  |  |  |  |  |  |  |  |  |  |  |  |  |  |  |  |  |  |  |  |  |  |  |  |  |  |  |  |  |  |  |  |  |  |  |  |  |  |  |  |  |  |  |  |  |  |  |  |  |  |  |  |  |  |  |  |  |  |  |  |  |  |  |  |  |  |  |  |  |  |  |  |  |  |  |  |  |  |  |  |  |  |  |  |  |  |  |  |  |  |  |  |  |  |  |  |  |  |  |  |  |  |  |  |  |  |  |  |  |  |  |  |  |  |  |  |  |  |  |  |  |  |  |  |  |  |  |  |  |  |  |  |  |  |  |  |  |  |  |  |  |  |  |  |  |  |  |  |  |  |  |  |  |  |  |  |  |  |  |  |  |  |  |  |  |  |  |  |  |  |  |  |  |  |  |  |  |  |  |  |  |  |  |  |  |  |  |  |
| --- | --- | --- | --- | --- | --- | --- | --- | --- | --- | --- | --- | --- | --- | --- | --- | --- | --- | --- | --- | --- | --- | --- | --- | --- | --- | --- | --- | --- | --- | --- | --- | --- | --- | --- | --- | --- | --- | --- | --- | --- | --- | --- | --- | --- | --- | --- | --- | --- | --- | --- | --- | --- | --- | --- | --- | --- | --- | --- | --- | --- | --- | --- | --- | --- | --- | --- | --- | --- | --- | --- | --- | --- | --- | --- | --- | --- | --- | --- | --- | --- | --- | --- | --- | --- | --- | --- | --- | --- | --- | --- | --- | --- | --- | --- | --- | --- | --- | --- | --- | --- | --- | --- | --- | --- | --- | --- | --- | --- | --- | --- | --- | --- | --- | --- | --- | --- | --- | --- | --- | --- | --- | --- | --- | --- | --- | --- | --- | --- | --- | --- | --- | --- | --- | --- | --- | --- | --- | --- | --- | --- | --- | --- | --- | --- | --- | --- | --- | --- | --- | --- | --- | --- | --- | --- | --- | --- | --- | --- | --- | --- | --- | --- | --- | --- | --- | --- | --- | --- | --- | --- | --- | --- | --- | --- | --- | --- | --- | --- | --- | --- | --- | --- | --- | --- | --- | --- | --- | --- | --- | --- | --- | --- | --- | --- | --- | --- | --- | --- | --- | --- | --- | --- | --- | --- | --- | --- | --- | --- | --- | --- | --- | --- | --- | --- | --- | --- | --- | --- | --- | --- | --- | --- | --- | --- | --- | --- | --- | --- | --- | --- | --- | --- | --- | --- | --- |
| Contig spectrum analysis results | Parameters:  |  |  |  |  |  |  |  |  |  |  |  |  | | --- | --- | --- | --- | --- | --- | --- | --- | --- | --- | --- | --- | | Contig spectrum: | [1021 17 3 0 0 0] || Avg. genome size: | 50000 bp || Avg. fragment length: | 650 bp || Min. overlap length: | 20 bp || Genotype range: | between 1 and 100000 || Precision: | 2 |  ---  Results:> The best results are those with the smallest error < Rank-abundance form: power > Structure model   |  |  |  | | --- | --- | --- | | - Error: | 3.7 | ? | | - Comment: | - | ? | | - Model parameter 1: | 0.73 | ? | | - Model parameter 2: | 0.026 | ? | | - Model equation: | ni = 0.026 i -0.73 | ? | | - Error minimization curve: |  | ? | | - Rank-abundance curve: |  | ? | | - Abundance values (%): | Here | ? |   > Diversity estimates   |  |  |  | | --- | --- | --- | | - Richness: | 8000 genotypes | ? | | - Evenness: | 0.9 | ? | | - Most abundant genotype: | 2.6 % of the community | ? | | - Shannon-Wiener index: | 8.1 nats | ? |  ---   Rank-abundance form: exponential > Structure model   |  |  |  | | --- | --- | --- | | - Error: | 24 | ? | | - Comment: | - | ? | | - Model parameter 1: | 0.004 | ? | | - Model parameter 2: | 0.004 | ? | | - Model equation: | ni = 0.004 e -0.004 i | ? | | - Error minimization curve: | Here | ? | | - Rank-abundance curve: | Here | ? | | - Abundance values (%): | Here | ? |   > Diversity estimates   |  |  |  | | --- | --- | --- | | - Richness: | 20001 genotypes | ? | | - Evenness: | 0.66 | ? | | - Most abundant genotype: | 0.4 % of the community | ? | | - Shannon-Wiener index: | 6.5 nats | ? |   ---   Rank-abundance form: logarithmic > Structure model   |  |  |  | | --- | --- | --- | | - Error: | 5.1 | ? | | - Comment: | - | ? | | - Model parameter 1: | 1.8 | ? | | - Model parameter 2: | 0.015 | ? | | - Model equation: | ni = 0.015 (log(i+1)) -1.8 | ? | | - Error minimization curve: | Here | ? | | - Rank-abundance curve: | Here | ? | | - Abundance values (%): | Here | ? |   > Diversity estimates   |  |  |  | | --- | --- | --- | | - Richness: | 1700 genotypes | ? | | - Evenness: | 0.97 | ? | | - Most abundant genotype: | 3 % of the community | ? | | - Shannon-Wiener index: | 7.2 nats | ? |   ---   Rank-abundance form: lognormal > Structure model   |  |  |  | | --- | --- | --- | | - Error: | 4.1 | ? | | - Comment: | - | ? | | - Model parameter 1: | 2.2 | ? | | - Model parameter 2: | 1.7e-06 | ? | | - Model equation: | ni = 1.7e-06 e 2.2 x(i) | ? | | - Error minimization curve: | Here | ? | | - Rank-abundance curve: | Here | ? | | - Abundance values (%): | Here | ? |   > Diversity estimates   |  |  |  | | --- | --- | --- | | - Richness: | 48000 genotypes | ? | | - Evenness: | 0.77 | ? | | - Most abundant genotype: | 2.7 % of the community | ? | | - Shannon-Wiener index: | 8.3 nats | ? |   ---   Rank-abundance form: niche\_preemption > Structure model   |  |  |  | | --- | --- | --- | | - Error: | 24 | ? | | - Comment: | - | ? | | - Model parameter 1: | 0.004 | ? | | - Model parameter 2: | 0 | ? | | - Model equation: | ni = 0.004 (1-0.004) i-1 | ? | | - Error minimization curve: | Here | ? | | - Rank-abundance curve: | Here | ? | | - Abundance values (%): | Here | ? |   > Diversity estimates   |  |  |  | | --- | --- | --- | | - Richness: | 10001 genotypes | ? | | - Evenness: | 0.71 | ? | | - Most abundant genotype: | 0.4 % of the community | ? | | - Shannon-Wiener index: | 6.5 nats | ? |   ---   Rank-abundance form: broken\_stick > Structure model   |  |  |  | | --- | --- | --- | | - Error: | 22 | ? | | - Comment: | - | ? | | - Model parameter 1: | 0 | ? | | - Model parameter 2: | 0 | ? | | - Model equation: | |  |  |  |  |  |  | | --- | --- | --- | --- | --- | --- | | ni = | 1 — M | M ∑ x = i 1 — x |  | | | | ? | | - Error minimization curve: | Here | ? | | - Rank-abundance curve: | Here | ? | | - Abundance values (%): | Here | ? |   > Diversity estimates   |  |  |  | | --- | --- | --- | | - Richness: | 1000 genotypes | ? | | - Evenness: | 0.94 | ? | | - Most abundant genotype: | 0.75 % of the community | ? | | - Shannon-Wiener index: | 6.5 nats | ? |   ---   Your results are still available 24 hours on this link! |
