# Supplementary material for: PHACCS, an online tool for estimating the structure and diversity of uncultured viral communities using metagenomic information
Source: BMC Bioinformatics. 2005 Mar 2;6:41. doi: 10.1186/1471-2105-6-41 (PMC555943; doi:10.1186/1471-2105-6-41)
Supplement: Additional File 1 — This file contains the script files part of PHACCS. These files are either standard text or picture files. [file 1471-2105-6-41-S1.zip › PHACCS_V101/html/phaccs/results/model-results-1872283102.htm]

Contig spectrum analysis results


|  |  |  |  |  |  |  |  |  |  |  |  |  |  |  |  |  |  |  |  |  |  |  |  |  |  |  |  |  |  |  |  |  |  |  |  |  |  |  |  |  |  |  |  |  |  |  |  |  |  |
| --- | --- | --- | --- | --- | --- | --- | --- | --- | --- | --- | --- | --- | --- | --- | --- | --- | --- | --- | --- | --- | --- | --- | --- | --- | --- | --- | --- | --- | --- | --- | --- | --- | --- | --- | --- | --- | --- | --- | --- | --- | --- | --- | --- | --- | --- | --- | --- | --- | --- |
| Contig spectrum analysis results | Parameters:  |  |  |  |  |  |  |  |  |  |  |  |  | | --- | --- | --- | --- | --- | --- | --- | --- | --- | --- | --- | --- | | Contig spectrum: | [1021 17 3 0 0 0] || Avg. genome size: | 50000 bp || Avg. fragment length: | 663 bp || Min. overlap length: | 20 bp || Genotype range: | between 1 and 100000 || Precision: | 3 |  ---  Results:> The best results are those with the smallest error <Rank-abundance form: power > Structure model   |  |  |  | | --- | --- | --- | | - Error: | 3.74 | ? | | - Comment: | - | ? | | - Model parameter 1: | 0.727 | ? | | - Model parameter 2: | 0.0253 | ? | | - Model equation: | ni = 0.0253 i -0.727 | ? | | - Error minimization curve: | Here | ? | | - Rank-abundance curve: | Here | ? | | - Abundance values (%): | Here | ? |   > Diversity estimates   |  |  |  | | --- | --- | --- | | - Richness: | 7990 genotypes | ? | | - Evenness: | 0.901 | ? | | - Most abundant genotype: | 2.53 % of the community | ? | | - Shannon-Wiener index: | 8.1 nats | ? |   ---   Your results are still available 24 hours on this link! |
