# Supplementary material for: PHACCS, an online tool for estimating the structure and diversity of uncultured viral communities using metagenomic information
Source: BMC Bioinformatics. 2005 Mar 2;6:41. doi: 10.1186/1471-2105-6-41 (PMC555943; doi:10.1186/1471-2105-6-41)
Supplement: Additional File 1 — This file contains the script files part of PHACCS. These files are either standard text or picture files. [file 1471-2105-6-41-S1.zip › PHACCS_V101/html/phaccs/glossary.htm]

Home


|  |  |
| --- | --- |
| GlossaryTerms employed and all you need to fill the forms | Abundance values:  This is a text file containting the relative rank-abundance values. The relative abundance values are listed according to their rank and expressed in percent of the total community. Average genome size:  The size of the genomes of a community is approximated by the average size (in bp) in PHACCS. This information can be determined experimentally by Pulse Field Gel Electrophoresis (PFGE). A typical genome size is 50 kb for phages in a marine environment. This value is used as default in the basic interface.  Average fragment length:  The length of the shotgun DNA fragments used in the sequence assembly process is approximated by the average length (in bp) in PHACCS. This information has to determined after trimming. The basic interface uses 650 bp as default for this parameter.  Comment:  The comment field tells the user if the data could not be modeled correctly (error's global minimum not found) and proposes him to redo the computation with an extended genotype range. Contig spectrum:  The contig spectrum is a vector containing the number of *q*-contigs (group of *q* overlapping sequences) found by assembly of DNA fragments. E.g. [104 6 2 0 0 0], means that there are 104 contigs of 1 sequence, 6 contigs of 2 sequences, 2 contigs of 3 sequences, and no contig containing 4 or more sequences. This data is the main input for modelling the communities with PHACCS.  Note that although the number of trailing zeros in the contig spectrum does not make a difference in the biological meaning, it affects the computation. Therefore to obtain the most accurate estimations you should make sure that you have enough trailing zeros when analysing your data. Be aware that adding zeros   Error:  The error in fit quantifies the difference between the predicted contig spectrum and the experimental one. It is calculated as the variance-weighted sum of squared deviations. Thus for the considered contig spectrum, the smaller the error, the better the model. The model with the smallest error is graphically highlighted in a colored box. Error minimization curve:  This graphics plots the error in function of the number of genotypes. The shape of this curve gives insights about how good the data can be modeled using a given rank-abundance form. Evenness:  This estimate quantifies the relative abundance of the genotypes in a community. The maximum value for the evenness is 1 and means that each genotype is as abundant as the other ones (flat rank-abundance curve). Genotype range:  PHACCS will try to find the best model (i.e., globally minimize the models' error in fit) within the range you specify. If PHACCS cannot find the error's global minimum, you should increase the search range. By default, the basic interface searches between 1 and 100,000 different genotypes. Note: Increasing the range value will also significantly increase the time needed for the analysis.  Graphics:  You can generate several graphics to represent your results. The error curve is the error in function of the numbebr of genotypes. The abundance curve (community structure curve) plots the abundance (in percent of the community) in function of the abundance rank. By default, the basic interface generates error and abundance curves. Mininimum overlap length:  The minimum overlap length (in bp) refers to the overlapping parameters used during assembly of the shotgun DNA fragments. This value has to be experimentally determined so that only fragments belonging to the same genotype overlap. For phage communities, we define overlapping sequences as having 98% identity on at least 20 bp with Sequencher. This value is used as default in the basic interface. Model equation:  This is the equation describing the structure of the community (its rank-abundance curve). Please refer to PHACCS' manuscript for more information. Model parameters:  "Model parameter 1" and "Model parameter 2" are the parameters for the best model given by a rank-abundance form. They are included in the model equation. Most abundant genotype:  This is the abundance of the most abundant genotype in the community, expressed in percent. Precision:  The precision refers to the number of significant figures of the number of genotypes. Since the number of genotypes is used to construct the community model and later on estimate its diversity, the precision also reflects the accuracy of the estimates. Note that the higher the precision, the higher the computation time. Rank-abundance curve:  This graphics represent the community structure by plotting the abundance of the genotypes in function of its rank (linear or logarithmic scale). Rank-abundance distribution:  PHACCS has to do an assumption about the rank-abundance distribution of the genotypes in a community. You can choose up to six abundance forms: power law, exponential, logarithmic, lognormal, niche preemption, and broken stick.The power law, lognormal, exponential and logarithmic distributions are empirical models, whereas the niche preemption and broken stick distributions are ecologically based on a division of the available ressources. By default, the basic interface uses all six models.Note that the broken stick and lognormal distributions take significantly more time to compute than the other ones.  Richness:  This estimate quantifies the number of different genotypes predicted in a community. Shannon-Wiener index:  This index quantify biodiversity as a combination of richness and evenness. The higher this index, the higher the diversity. |
