# Supplementary material for: PHACCS, an online tool for estimating the structure and diversity of uncultured viral communities using metagenomic information
Source: BMC Bioinformatics. 2005 Mar 2;6:41. doi: 10.1186/1471-2105-6-41 (PMC555943; doi:10.1186/1471-2105-6-41)
Supplement: Additional File 1 — This file contains the script files part of PHACCS. These files are either standard text or picture files. [file 1471-2105-6-41-S1.zip › PHACCS_V101/html/phaccs/error.htm]

Error


|  |  |
| --- | --- |
| Error | An error has occured!*\_\_\_error\_\_\_* Please contact the webmaster if the problem persists. |
