# Supplementary material for: PHACCS, an online tool for estimating the structure and diversity of uncultured viral communities using metagenomic information
Source: BMC Bioinformatics. 2005 Mar 2;6:41. doi: 10.1186/1471-2105-6-41 (PMC555943; doi:10.1186/1471-2105-6-41)
Supplement: Additional File 1 — This file contains the script files part of PHACCS. These files are either standard text or picture files. [file 1471-2105-6-41-S1.zip › PHACCS_V101/html/phaccs/home.htm]

Home


|  |  |
| --- | --- |
| PHACCSis an online bioinformatic tool to assess the biodiversity of uncultured viral communities | Contig spectrum analysis:PHACCS models the structure and estimates the diversity of uncultured viral communities, based on the contig spectrum, a metagenomic information obtained from environmental samples, and using a modified version of the Lander-Waterman algorithm. - Basic interface - Advanced interface ---      Resource:If it is your first time using PHACCS, I strongly suggest you consult the resource section.     It gives access to more information about PHACCS, how it works and how to use it... ---      Program:Program presentation, change log, download, ... - December 2004 - January 2005 / Finalization of the program and its interface - September 2004 / New improved website More... |
