# Supplementary material for: PHACCS, an online tool for estimating the structure and diversity of uncultured viral communities using metagenomic information
Source: BMC Bioinformatics. 2005 Mar 2;6:41. doi: 10.1186/1471-2105-6-41 (PMC555943; doi:10.1186/1471-2105-6-41)
Supplement: Additional File 1 — This file contains the script files part of PHACCS. These files are either standard text or picture files. [file 1471-2105-6-41-S1.zip › PHACCS_V101/html/phaccs/modeladvanced.htm]

Contig spectrum analysis (advanced interface)


|  |  |  |  |  |  |  |  |  |  |  |  |  |  |  |  |  |  |  |  |  |  |  |  |  |  |  |  |  |
| --- | --- | --- | --- | --- | --- | --- | --- | --- | --- | --- | --- | --- | --- | --- | --- | --- | --- | --- | --- | --- | --- | --- | --- | --- | --- | --- | --- | --- |
| Contig spectrum analysis  Advanced interface | The advanced interface is for the custom analysis of any viral community and predictions about its:- **structure**: best relative abundance functional form and model's equation, and - **diversity**: richness, evenness, Shannon-Wiener index, relative abundance of the most abundant genotype. ---       > Data      |  |  |  |  |  |  |     | --- | --- | --- | --- | --- | --- |     | - Contig spectrum: | |  |  |  | | --- | --- | --- | | [ |  | ] | | ? |     | - Avg. genome size (bp): |  | ? |     | - Avg. fragment length (bp): |  | ? |     | - Min. overlap length (bp): |  | ? |      > Computation      |  |  |  |     | --- | --- | --- |     | - Rank-abundance distribution: | Power Exponential  Logarithmic Lognormal  Niche Preemption Broken Stick | ? |     | - Genotype range: | from  to | ? |     | - Precision: | 2345 | ? |     | - Graphics: | Error curve  Abundance curve  Abundance curve (log scale) | ? |        *Note: Depending on your analysis the computation can take a while. Please be patient!*        ---       Switch to the basic interface if you don't know what to put in these fields. |
