# Supplementary material for: PHACCS, an online tool for estimating the structure and diversity of uncultured viral communities using metagenomic information
Source: BMC Bioinformatics. 2005 Mar 2;6:41. doi: 10.1186/1471-2105-6-41 (PMC555943; doi:10.1186/1471-2105-6-41)
Supplement: Additional File 1 — This file contains the script files part of PHACCS. These files are either standard text or picture files. [file 1471-2105-6-41-S1.zip › PHACCS_V101/html/phaccs/model-parameters.htm]

# Parameters:

|  |  |  |  |  |  |  |  |  |  |  |  |
| --- | --- | --- | --- | --- | --- | --- | --- | --- | --- | --- | --- |
| Contig spectrum: | \_\_\_goalcontig\_\_\_ || Avg. genome size: | \_\_\_genlength\_\_\_ bp || Avg. fragment length: | \_\_\_truefraglength\_\_\_ bp || Min. overlap length: | \_\_\_overlaplength\_\_\_ bp || Genotype range: | between \_\_\_first\_\_\_ and \_\_\_last\_\_\_ || Precision: | \_\_\_significant\_\_\_ |

---

# Results:

> The best results are those with the smallest error <
