# Supplementary material for: PHACCS, an online tool for estimating the structure and diversity of uncultured viral communities using metagenomic information
Source: BMC Bioinformatics. 2005 Mar 2;6:41. doi: 10.1186/1471-2105-6-41 (PMC555943; doi:10.1186/1471-2105-6-41)
Supplement: Additional File 1 — This file contains the script files part of PHACCS. These files are either standard text or picture files. [file 1471-2105-6-41-S1.zip › PHACCS_V101/html/phaccs/model-results.htm]

### Rank-abundance form: \_\_\_scenario\_\_\_ > Structure model | | | | | --- | --- | --- | | - Error: | \_\_\_fit\_\_\_ | ? | | - Comment: | \_\_\_comment\_\_\_ | ? | | - Model parameter 1: | \_\_\_parameter\_\_\_ | ? | | - Model parameter 2: | \_\_\_parameter2\_\_\_ | ? | | - Model equation: | \_\_\_equation\_\_\_ | ? | | - Error minimization curve: | \_\_\_errorgraphic\_\_\_ | ? | | - Rank-abundance curve: | \_\_\_abundancegraphic\_\_\_ | ? | | - Abundance values (%): | \_\_\_rankabundancevalues\_\_\_ | ? | > Diversity estimates | | | | | --- | --- | --- | | - Richness: | \_\_\_species\_\_\_ genotypes | ? | | - Evenness: | \_\_\_evenness\_\_\_ | ? | | - Most abundant genotype: | \_\_\_abundance\_\_\_ % of the community | ? | | - Shannon-Wiener index: | \_\_\_shannon\_\_\_ nats | ? | ---
