# Supplementary material for: PHACCS, an online tool for estimating the structure and diversity of uncultured viral communities using metagenomic information
Source: BMC Bioinformatics. 2005 Mar 2;6:41. doi: 10.1186/1471-2105-6-41 (PMC555943; doi:10.1186/1471-2105-6-41)
Supplement: Additional File 1 — This file contains the script files part of PHACCS. These files are either standard text or picture files. [file 1471-2105-6-41-S1.zip › PHACCS_V101/html/phaccs/program.htm]

History


|  |  |  |  |  |
| --- | --- | --- | --- | --- |
| ProgramTechnical information, change log, license & download | PHACCS:PHACCS (PHAge Communities from Contig Spectrum) was originally developped by myself (Florent Angly) in 2004 for the Rohwer's lab at San Diego State University (SDSU) as a diploma project for my school, the Ecole Sup�rieure de Strabourg (ESBS). |  |  |  | | --- | --- | --- | |  |  |  |   Basically, PHACCS rely on a mathematical core based on Matlab scripts (originally written by ) implementing a modified Lander-Waterman algorithm. Cgi-Perl scripts are used as a bridge to a web interface. ---  License:PHACCS is licensed under the GNU General Public License. A copy of it is located Here. ---  Download:You will be able to download an archive containing the whole PHACCS program here soon... ---  History: - December 2004 - January 2005      / Finalization of the program and its interface - September 2004      / New improved website - July 2004      / First online working version - January-Juny 2004      / Development of PHACCS |
