# Supplementary material for: PHACCS, an online tool for estimating the structure and diversity of uncultured viral communities using metagenomic information
Source: BMC Bioinformatics. 2005 Mar 2;6:41. doi: 10.1186/1471-2105-6-41 (PMC555943; doi:10.1186/1471-2105-6-41)
Supplement: Additional File 1 — This file contains the script files part of PHACCS. These files are either standard text or picture files. [file 1471-2105-6-41-S1.zip › PHACCS_V101/html/phaccs/resource.htm]

Resource


|  |  |
| --- | --- |
| ResourceSome help to use and understand PHACCS | GlossaryA definition of the terms employed on the website and explanations on how to fill the forms. Go >>> ---  PublicationA manuscript has been submitted to BMC Bioinformatics. It describes how PHACCS works and presents a case study of 4 phage communities. *It will be available here as soon as it is accepted!* ---  Poster presentationPHACCS has presented at several occasions like the San Diego Microbiology Group (SDMG) 2004 Meeting or for my graduation at the Ecole Sup�rieure de Strabourg (ESBS). Go >>> |
