# Supplementary figures and images for: PHACCS, an online tool for estimating the structure and diversity of uncultured viral communities using metagenomic information
Source: BMC Bioinformatics. 2005 Mar 2;6:41. doi: 10.1186/1471-2105-6-41 (PMC555943; doi:10.1186/1471-2105-6-41)

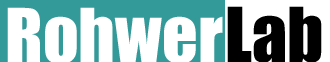

Supplement: Additional File 1 — This file contains the script files part of PHACCS. These files are either standard text or picture files. [file 1471-2105-6-41-S1.zip › PHACCS_V101/html/phaccs/pictures/rohwerlab-logo.gif]

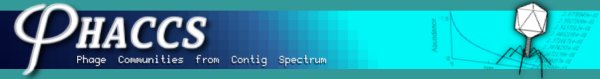

Supplement: Additional File 1 — This file contains the script files part of PHACCS. These files are either standard text or picture files. [file 1471-2105-6-41-S1.zip › PHACCS_V101/html/phaccs/pictures/logo-phaccs.jpg]

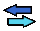

Supplement: Additional File 1 — This file contains the script files part of PHACCS. These files are either standard text or picture files. [file 1471-2105-6-41-S1.zip › PHACCS_V101/html/phaccs/pictures/switch-big.gif]

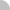

Supplement: Additional File 1 — This file contains the script files part of PHACCS. These files are either standard text or picture files. [file 1471-2105-6-41-S1.zip › PHACCS_V101/html/phaccs/pictures/upperright.png]

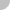

Supplement: Additional File 1 — This file contains the script files part of PHACCS. These files are either standard text or picture files. [file 1471-2105-6-41-S1.zip › PHACCS_V101/html/phaccs/pictures/lowerright.png]

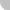

Supplement: Additional File 1 — This file contains the script files part of PHACCS. These files are either standard text or picture files. [file 1471-2105-6-41-S1.zip › PHACCS_V101/html/phaccs/pictures/lowerleft.png]

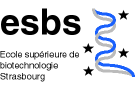

Supplement: Additional File 1 — This file contains the script files part of PHACCS. These files are either standard text or picture files. [file 1471-2105-6-41-S1.zip › PHACCS_V101/html/phaccs/pictures/esbs-logo.gif]

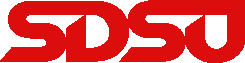

Supplement: Additional File 1 — This file contains the script files part of PHACCS. These files are either standard text or picture files. [file 1471-2105-6-41-S1.zip › PHACCS_V101/html/phaccs/pictures/sdsu-logo.gif]

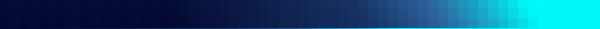

Supplement: Additional File 1 — This file contains the script files part of PHACCS. These files are either standard text or picture files. [file 1471-2105-6-41-S1.zip › PHACCS_V101/html/phaccs/pictures/banner.gif]

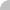

Supplement: Additional File 1 — This file contains the script files part of PHACCS. These files are either standard text or picture files. [file 1471-2105-6-41-S1.zip › PHACCS_V101/html/phaccs/pictures/upperleft.png]

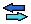

Supplement: Additional File 1 — This file contains the script files part of PHACCS. These files are either standard text or picture files. [file 1471-2105-6-41-S1.zip › PHACCS_V101/html/phaccs/pictures/switch.gif]

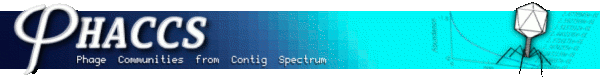

Supplement: Additional File 1 — This file contains the script files part of PHACCS. These files are either standard text or picture files. [file 1471-2105-6-41-S1.zip › PHACCS_V101/html/phaccs/pictures/logo-phaccs.gif]

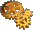

Supplement: Additional File 1 — This file contains the script files part of PHACCS. These files are either standard text or picture files. [file 1471-2105-6-41-S1.zip › PHACCS_V101/html/phaccs/pictures/gears.gif]

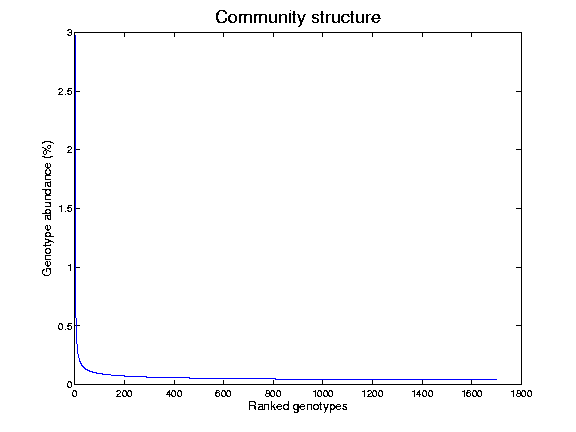

Supplement: Additional File 1 — This file contains the script files part of PHACCS. These files are either standard text or picture files. [file 1471-2105-6-41-S1.zip › PHACCS_V101/html/phaccs/results/abundance-logarithmic-7121814798.gif]

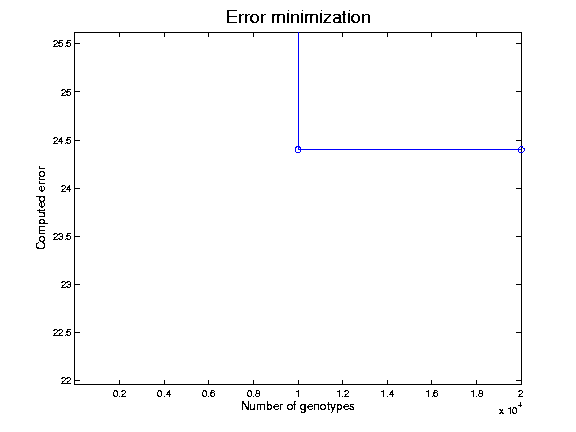

Supplement: Additional File 1 — This file contains the script files part of PHACCS. These files are either standard text or picture files. [file 1471-2105-6-41-S1.zip › PHACCS_V101/html/phaccs/results/error-niche_preemption-7121814798.gif]

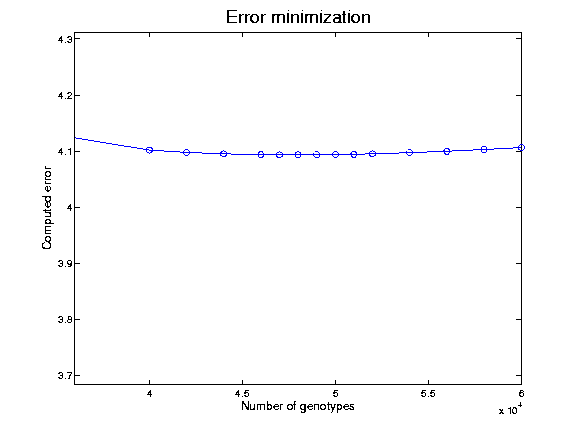

Supplement: Additional File 1 — This file contains the script files part of PHACCS. These files are either standard text or picture files. [file 1471-2105-6-41-S1.zip › PHACCS_V101/html/phaccs/results/error-lognormal-7121814798.gif]

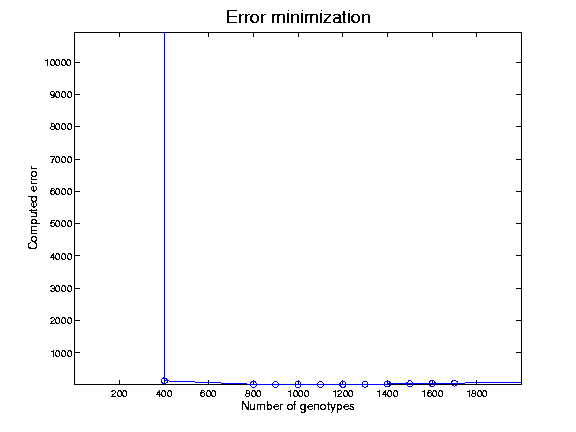

Supplement: Additional File 1 — This file contains the script files part of PHACCS. These files are either standard text or picture files. [file 1471-2105-6-41-S1.zip › PHACCS_V101/html/phaccs/results/error-broken_stick-7121814798.gif]

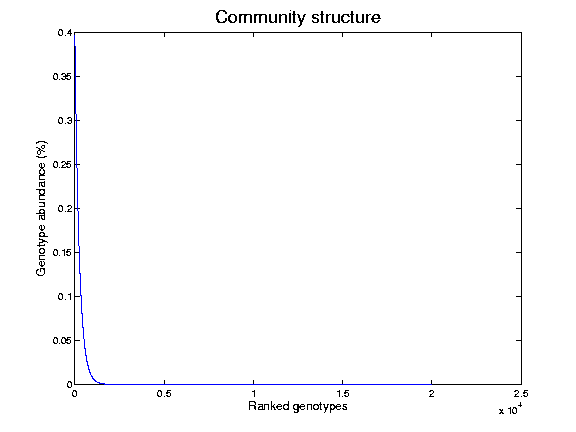

Supplement: Additional File 1 — This file contains the script files part of PHACCS. These files are either standard text or picture files. [file 1471-2105-6-41-S1.zip › PHACCS_V101/html/phaccs/results/abundance-exponential-7121814798.gif]

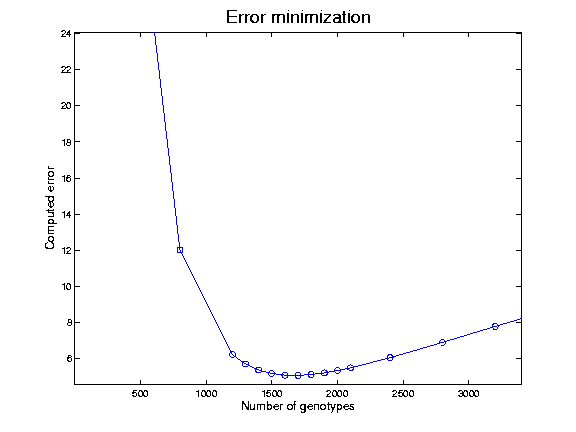

Supplement: Additional File 1 — This file contains the script files part of PHACCS. These files are either standard text or picture files. [file 1471-2105-6-41-S1.zip › PHACCS_V101/html/phaccs/results/error-logarithmic-7121814798.gif]

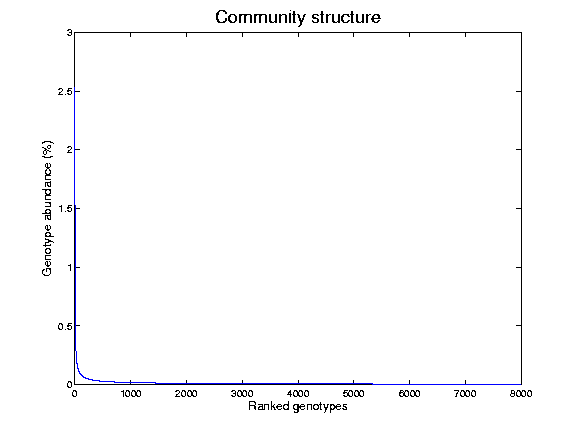

Supplement: Additional File 1 — This file contains the script files part of PHACCS. These files are either standard text or picture files. [file 1471-2105-6-41-S1.zip › PHACCS_V101/html/phaccs/results/abundance-power-1872283102.gif]

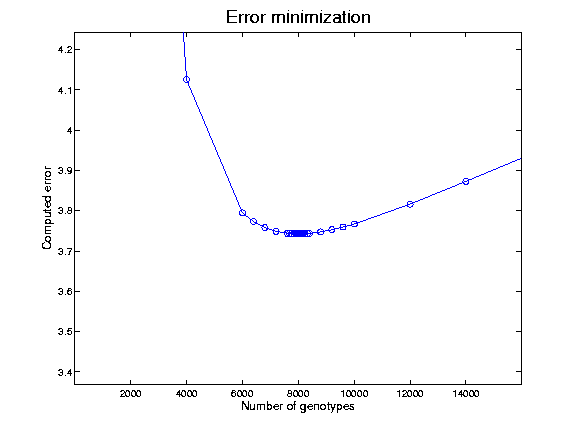

Supplement: Additional File 1 — This file contains the script files part of PHACCS. These files are either standard text or picture files. [file 1471-2105-6-41-S1.zip › PHACCS_V101/html/phaccs/results/error-power-1872283102.gif]

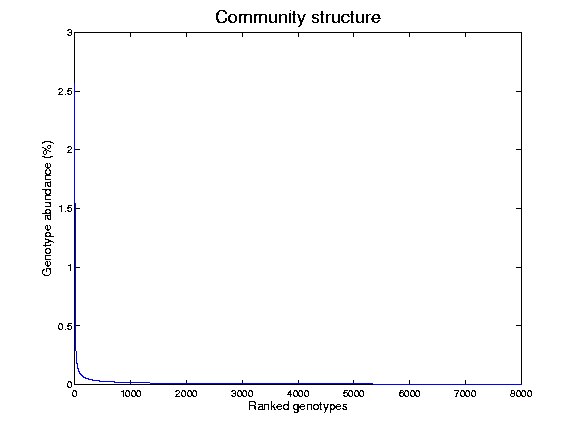

Supplement: Additional File 1 — This file contains the script files part of PHACCS. These files are either standard text or picture files. [file 1471-2105-6-41-S1.zip › PHACCS_V101/html/phaccs/results/abundance-power-7121814798.gif]

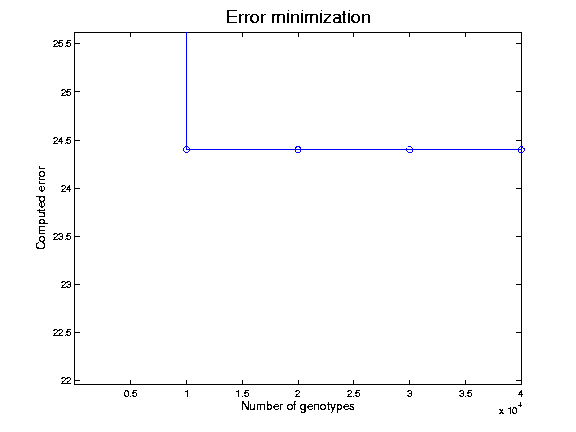

Supplement: Additional File 1 — This file contains the script files part of PHACCS. These files are either standard text or picture files. [file 1471-2105-6-41-S1.zip › PHACCS_V101/html/phaccs/results/error-exponential-7121814798.gif]

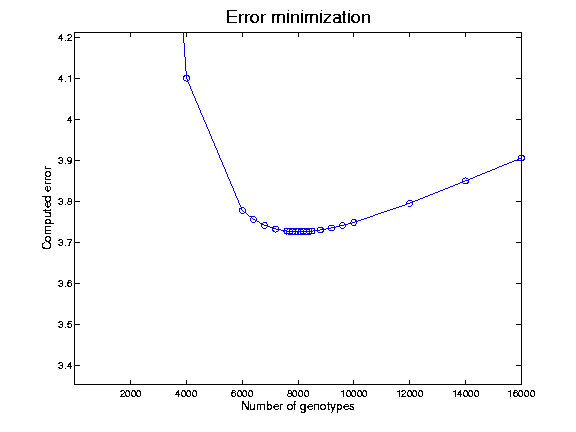

Supplement: Additional File 1 — This file contains the script files part of PHACCS. These files are either standard text or picture files. [file 1471-2105-6-41-S1.zip › PHACCS_V101/html/phaccs/results/error-power-7121814798.gif]

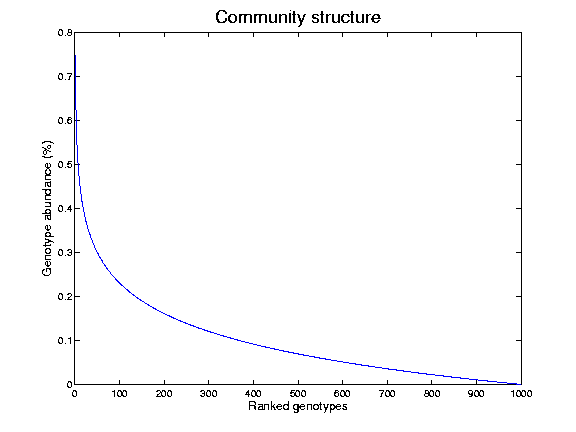

Supplement: Additional File 1 — This file contains the script files part of PHACCS. These files are either standard text or picture files. [file 1471-2105-6-41-S1.zip › PHACCS_V101/html/phaccs/results/abundance-broken_stick-7121814798.gif]
